# Metabotropic glutamate receptor 5 knockout reduces cognitive impairment and pathogenesis in a mouse model of Alzheimer's disease

**DOI:** 10.1186/1756-6606-7-40

**Published:** 2014-05-29

**Authors:** Alison Hamilton, Jessica L Esseltine, Rebecca A DeVries, Sean P Cregan, Stephen S G Ferguson

**Affiliations:** 1 The J. Allyn Taylor Centre for Cell Biology, Robarts Research Institute, The University of Western Ontario, 100 Perth Dr, London, Ontario N6A 5 K8, Canada

**Keywords:** Alzheimer’s disease, APPswe/PS1ΔE9, mGluR5, Beta amyloid, FMRP, Learning and memory

## Abstract

**Background:**

Alzheimer’s disease (AD) pathology occurs in part as the result of excessive production of β-amyloid (Aβ). Metabotropic glutamate receptor 5 (mGluR5) is now considered a receptor for Aβ and consequently contributes to pathogenic Aβ signaling in AD.

**Results:**

Genetic deletion of mGluR5 rescues the spatial learning deficits observed in APPswe/PS1ΔE9 AD mice. Moreover, both Aβ oligomer formation and Aβ plaque number are reduced in APPswe/PS1ΔE9 mice lacking mGluR5 expression. In addition to the observed increase in Aβ oligomers and plaques in APPswe/PS1ΔE9 mice, we found that both mTOR phosphorylation and fragile X mental retardation protein (FMRP) expression were increased in these mice. Genetic deletion of mGluR5 reduced Aβ oligomers, plaques, mTOR phosphorylation and FMRP expression in APPswe/PS1ΔE9 mice.

**Conclusions:**

Thus, we propose that Aβ activation of mGluR5 appears to initiate a positive feedback loop resulting in increased Aβ formation and AD pathology in APPswe/PS1ΔE9 mice via mechanism that is regulated by FMRP.

## Introduction

Alzheimer’s disease (AD) is the most prevalent neurodegenerative illness in older adults and has a characteristic neuropathology that includes plaques comprised of β amyloid (Aβ) and tangles of hyperphosphorylated tau [1,2]. Aβ42 is formed by the sequential proteolytic cleavage of the amyloid precursor protein (APP), by β- and γ-secretases, via what is referred to as the amyloidogenic pathway and are commonly found as soluble oligomers and fibrillar plaques [3]. A considerable body of evidence suggests that soluble Aβ oligomers are the predominant neurotoxic species of Aβ, with the Aβ42 fragment being found to be particularly potent [4,5]. Aβ42 oligomers exert their toxic effects by binding to neuronal synapses, causing disruption to normal synaptic signaling, which often leads to neuronal death [6,7]. However, the precise receptors to which Aβ42 binds remain very controversial.

Glutamate is the primary excitatory neurotransmitter in the brain and activates both ionotropic glutamate receptors and G protein-coupled metabotropic glutamate receptors (mGluRs) [8]. Alterations in normal mGluR5 signaling is associated with the autism spectrum disorder, fragile X syndrome, and a number of neurodegenerative diseases that include: Huntington’s disease, Parkinson’s disease and AD [9-17]. mGluR5 couples to the heterotrimeric G protein Gα_q/11_, which activates phospholipase C resulting in increased inositol-1,4,5-triphosphate formation and the release of Ca^2+^ from intracellular stores [3,12]. Recently, it has been demonstrated that mGluR5 also serves as the receptor for both cellular prion protein (PrP^c^) and Aβ42, which activate mGluR5 to release Ca^2+^ from intracellular stores [17-20].

It is now well recognized that mGluR5 regulates the activity of the fragile X mental retardation protein (FMRP), a RNA binding protein that functions to repress protein synthesis at synapses [21-25]. The activation of mGluR5 results in FMRP-dependent increases in both APP and FMRP expression [16,26]. This increase in APP expression results in the augmented secretion of both toxic Aβ42 oligomers and the non-toxic soluble APP (sAPPα) fragment [16,24]. Thus, although the precise mechanism remains unclear, this data suggests that, in addition to an established role in fragile X syndrome, mGluR5 and FMRP likely contribute directly to AD pathogenesis [16,27].

In the present study, we assess the role for mGluR5 in AD pathogenesis by crossing the APPswe/PS1∆E9 double transgenic mouse model of AD with mGluR5 null mice. We find that the genetic deletion of mGluR5 reverses the spatial memory deficits observed in APPswe/PS1∆E9 mice. FMRP protein expression is also increased APPswe/PS1∆E9 mice and following the genetic deletion of mGluR5 FMRP expression is reduced to wild-type levels and results in the significant decrease of aggregated β-amyloid levels and β-amyloid plaque number in APPswe/PS1∆E9 mice. These studies provide evidence for a central role of mGluR5 in AD pathogenic mechanisms that are associated with increased Aβ.

## Results

### Genetic deletion of mGluR5 rescues spatial memory deficits in APPswe/PS1ΔE9 mice

**Figure 1:**
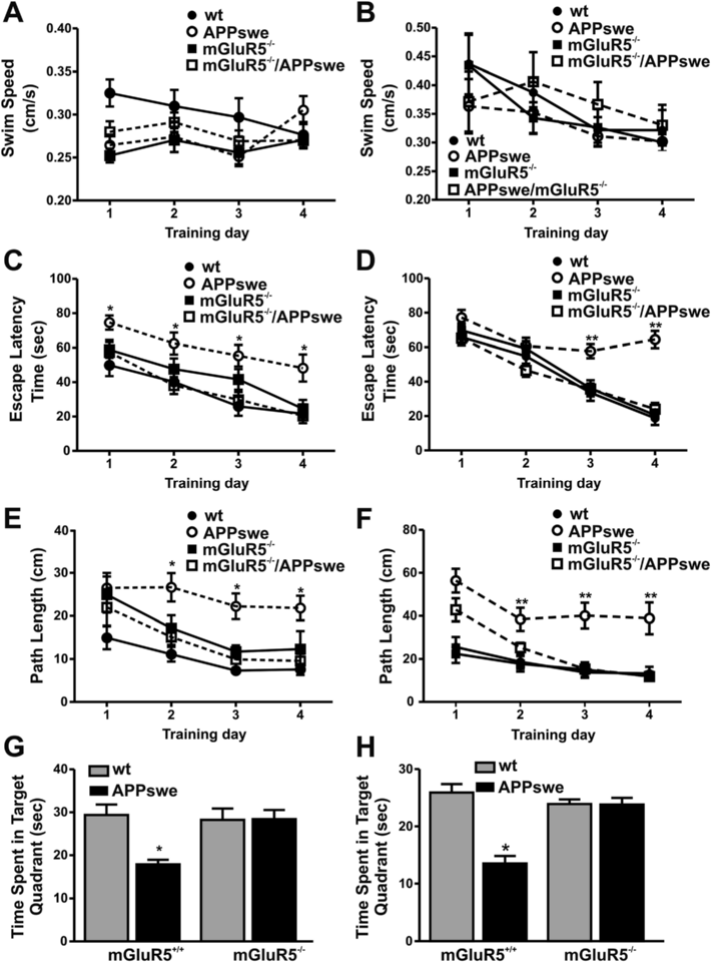
**Genetic deletion of mGluR5 rescues spatial memory deficits in APPswe/PS1ΔE9 mice at 9 and 12 months of age.** Shown are the swim speeds at **(A)** 9 and **(B)** 12 months of age, escape latencies, at **(C)** 9 and **(D)** 12 months of age, and path lengths **(E)** at 9 and **(F)** 12 months of age for wild-type (wt) (n = 16), APPswe/PSΔE9 (APPswe) (n = 11), mGluR5-/- (n = 14), and APPswe/mGluR5-/- (n = 14) mice recorded during the acquisition phase in the Morris Water Maze test for spatial learning. Shown is the time that wt, APPswe, mGluR5-/-, and APPswe/ mGluR5-/- mice spent in the target quadrant during the probe trial at **(G)** 9 and **(H)** 12 months of age. Data represent the mean ± SEM. Statistically significant * (p < 0.01) and ** (p < 0.001) differences between mice groups when compared to wild-type mice. Statistical significance was assessed by repeated measures ANOVA.

Given the potential role for mGluR5 as a receptor for Aβ [17-20], we crossed mGluR5 knockout mice with APPswe/PS1∆E9 mice. The double transgenic APPswe/PS1∆E9 mice were previously found to exhibit impaired cognitive function as early as 7 months of age [28-30]. Therefore, our initial studies tested whether the genetic deletion of mGluR5 improved spatial memory deficits in APPswe/PS1∆E9 mice at both nine and twelve months of age in the Morris Water Maze test. Mice were exposed to the maze for sixteen 90 s trials over 4 days during the acquisition phase (4 trials per day), followed by a 60 s probe trial on day 5. The swimming ability for all four mouse genotypes was consistent and indistinguishable at both nine and twelve months of age (Figure 1A and B). However, repeated measures analysis of variance indicated that spatial learning of 9 month old mice (Figure 1C and E), as measured by either the escape latency or swim path length, varied as a function of the genotype, F(3,41) = 4.83, and 11.20, p’s < 0.01, respectively. Precisely the same profile was apparent in mice at 12 months of age (Figure 1D and F). Follow-up Bonferonni corrected t tests confirmed that performance of APPswe/PS1∆E9 mice was significantly impaired when compared to wild-type and mGluR5^-/-^ mice. However, the genetic deletion of mGluR5 rescued the spatial learning deficit in APPswe/PS1∆E9 mice at both ages. The genotype differences were consistent and the interaction between Strain and Days did not reach statistical significance.

During the probe trial on day 5, in which the platform was removed, the genotypes varied appreciably with respect to the time spent in the target quadrant, F(3,41) = 20.62 and 21.66, p ‘s < 0.001 for mice tested at 9 and 12 months respectively. The follow-up tests confirmed that APPswe/PS1∆E9 mice spent less time in the target quadrant relative to all other genotypes at either nine or twelve months of age, but that the loss of mGluR5 expression improved APPswe/PS1∆E9 mouse behaviour (Figure 1G and H). The time that the mice spent in each quadrant of the pool is shown in Additional file 1: Figure S1. Thus, our observation that the genetic deletion of mGluR5 resulted in significantly improved spatial temporal memory in APPswe/PS1∆E9 mice was consistent with a recent report that pharmacological blockade of mGluR5 improved behavioural deficits in APPswe/PS1∆E9 mice [17].

### Effect of APPswe/PS1ΔE9 mutation on the locomotor, anxiety and exploratory behavioural phenotypes of mGluR5^-/-^ mice

**Figure 2:**
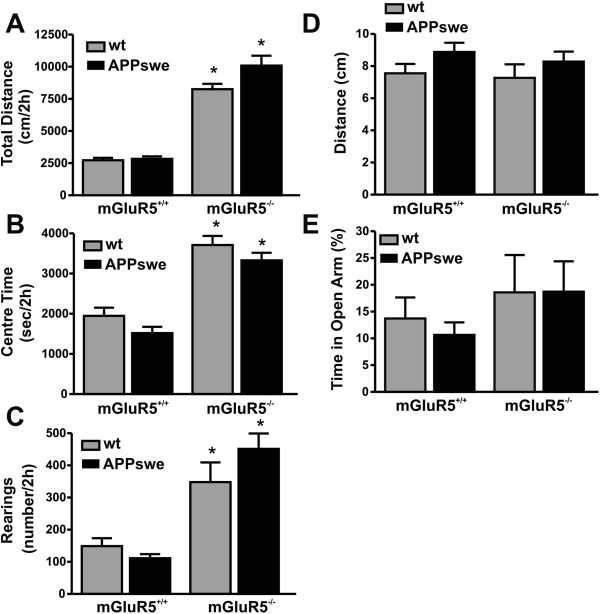
**Genetic deletion of mGluR5 increases locomotor, anxiety and explorative behaviours in wild-type and APPswe/PS1ΔE9 mice at 12 months of age.** Graphs show **(A)** the total distance traveled, **(B)** the time spent in the centre quadrant of the open field and **(C)** number of rearings for wild-type (wt) and APPswe/PS1ΔE9 (APPswe) mice on either mGluR5+/+ or mGluR5-/- genetic backgrounds. **(D)** Show is the total distance traveled in the elevated plus maze. **(E)** Shown is the percentage of time spent in the open arm of the elevated plus maze. The number of animals tested: n = 16 for wt, n = 11 APPswe, n = 14 for mGluR5-/- and n = 14 for APPswe/mGluR5-/- mice. Data represent the mean ± SEM. The * indicates statistically significant (p < 0.05) differences between mice groups as compared with wild-type mice.

We previously showed that mGluR5^-/-^ mice exhibit increased locomotor activity that is reversed in the Q111 knock-in mutant huntingtin genetic background [12]. Moreover, APPswe/PS1∆E9 mice were previously shown to exhibit impaired exploratory behavior [28,29]. Therefore, we wanted to ascertain that double transgenic expression of APPswe and PS1∆E9 does not alter changes in the locomotor (distance traveled), anxiety (centre time) and exploratory (number of rearing events) behaviours induced by mGluR5 deletion in twelve month old wild-type and APPswe/PS1∆E9 mice lacking mGluR5 expression over a 2 h period in an open field arena. We found no differences in the locomotor, centre time and exploratory behaviours of APPswe/PS1∆E9 mice when compared to wild type controls (Figure 2A-C). However, the genetic deletion of mGluR5 resulted in locomotor hyperactivity, reduced centre time in the open field, as well as increased exploratory behaviours in both APPswe/PS1∆E9 mice and wild type controls (Figure 2A-C). However, we did not find a statistically significant increase in anxiety behaviour in mGluR5 knockout mice in the elevated plus maze (Figure 2D and E). Thus, double transgenic expression of APPswe and PS1∆E9 does not alter the changes in locomotor, anxiety and exploratory behaviour that occur as the consequence of mGluR5 deletion.

### Genetic deletion of mGluR5 reduces Aβ in APPswe/PS1ΔE9 mice

**Figure 3:**
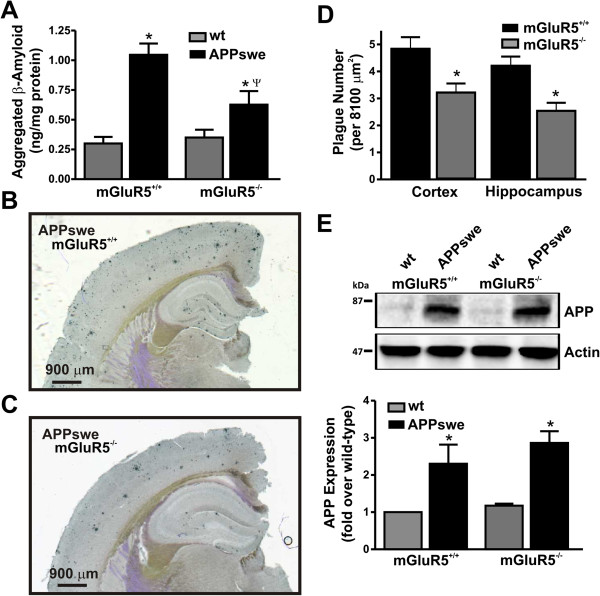
**Aβ formation and plaque number is reduced in 12 month old APPswe/PS1ΔE9 mice following the genetic deletion of mGluR5.** Shown in **(A)** is the concentration of whole brain β amyloid oligomer (ng/mg protein) concentrations in 12 month old wild-type (wt) and APPswe/PS1ΔE9 (APPswe) mice on either mGluR5+/+or mGluR5-/- genetic backgrounds. Data represents the mean ± SEM of four independent experiments. The * indicates statistically significant (p < 0.05) differences between β amyloid oligomer concentrations in APPswe/PS1ΔE9 mouse brains as compared with mice that do not express APPswe/PS1ΔE9. The ^ψ^ indicates statistically significant differences between β amyloid oligomer concentrations in APPswe mice that express mGluR5 as compared to APPswe mice that lack mGluR5 expression. Also shown are representative images of Aβ plaques in the cortex and hippocampus of 12 month old coronal brain slices from **(B)** APPsweand **(C)** APPswe/mGluR5-/- mice. Scale bar is 900 μm. **(D)** The graph shows the number of plaques counted 8100 μm^2^ regions of interests in the cortex and hippocampus in coronal brain slices from 12 month old APPswe and APPswe/mGluR5-/- mice. **(E)** Representative immunoblot and quantification of APP expression in wt and APPSwe mice either expressing or lacking mGluR5 expression. Data represents the mean ± SEM of four independent experiments. The * indicates statistically significant (p < 0.05) differences between APPswe expressing mGluR5 as are compared with APPswe lacking mGluR5 expression.

Given that mGluR5 deletion improved cognition, we sought to examine the effect on Aβ production and deposition. The deposition of Aβ in APPswe/PS1∆E9 mice occurs as early as 3 months of age [29-31]. Therefore, to assess the role of mGluR5 on Aβ oligomer load, whole brains from 12 month old mice were analyzed for Aβ oligomers using a sandwich ELISA assay. We found a 3.7 ± 0.5 fold increase in Aβ oligomers in APPswe/PS1∆E9 mice when compared to wild-type controls, this increase in Aβ oligomers in APPswe/PS1∆E9 mice was reduced to 1.2 ± 0.2 fold following the genetic deletion of mGluR5 (Figure 3A). This represented a 41 ± 6% reduction in Aβ oligomers in APPswe/PS1∆E9 mice lacking mGluR5 expression when compared to APPswe/PS1∆E9 mice that express mGluR5. We also examined whether the number of Aβ plaques were altered in APPswe/PS1∆E9 mice lacking mGluR5 to rule out the possibility that Aβ oligomers were simply being converted more efficiently to plaques to reduce cognitive decline. When quantified we found that Aβ plaque number was also reduced in the cortex and hippocampus of coronal brain slices, with the Aβ plaque load reduced by 34% and 40% in cortex and hippocampus, respectively (Figure 3B-D). Because APP was transgenically (ectopically) overexpressed in APPswe/PS1∆E9 mice, we tested whether the reduction of Aβ following the genetic deletion of mGluR5 was associated with reduced APP expression in whole brain lysates derived from 12 month old wild type and APPswe/PS1∆E9 mice that either expressed or lacked mGluR5. We found that the genetic deletion of mGluR5 had no apparent effect on the transgenic APP expression in double transgenic APPswe/PS1∆E9 mice (Figure 3E). Thus, the expression of mGluR5 appeared to influence the cleavage of APP towards an amyloidogenic pathway in the APPswe/PS1∆E9 double transgenic mouse model, and mGluR5 deletion functions to reduce both Aβ oligomer formation and plaque number in APPswe/PS1∆E9 mice.

### mGluR5 cell surface expression and InsP signaling in APPswe/PS1ΔE9 mice

**Figure 4:**
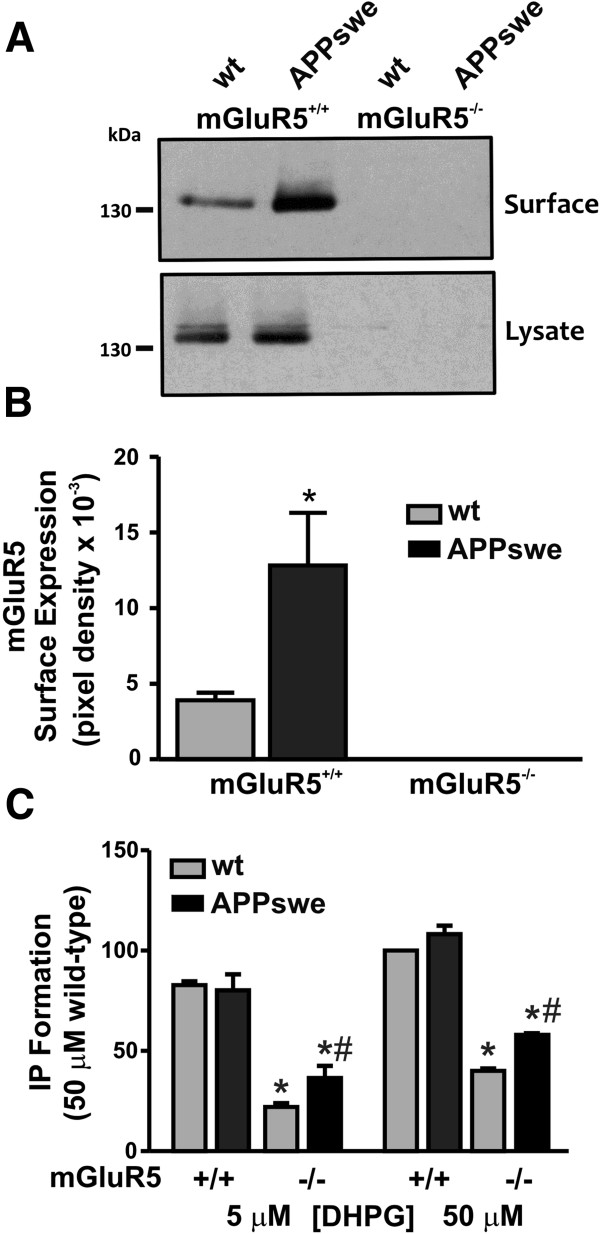
**Increased mGluR5 cell surface expression does not alter InsP signaling in 12 month old APPswe/PS1ΔE9 mice*. *(A)** Shown is a representative immunoblot showing increased cell surface expression, but not total cellular expression, of mGluR5 in brain lysates derived from 12 month wild-type (wt) versus APPswe/PS1ΔE9 (APPswe) mice. Loss of mGluR5 expression following mGluR5 knockout is also shown for both mouse lines. **(B)** Graph shows the quantification of the relative cell surface expression of mGluR5 in wt versus APPswe mice at 12 months of age. Data represents the mean ± SEM of four independent experiments. The * indicates statistically significant (P < 0.05) differences between in APPswe mice, as compared to wt mice (P < 0.05). Shown in **(C)** is mGluR1/5-stimulated InsP formation in response to either 5 μM or 50 μM DHPG for 15 min at 37°C in cortical prisms derived from either wt or APPswe mice, that either express mGluR5 (+/+) or do not express mGluR5 (-/-). The data represents the means ± SEM of four independent experiments, and is expressed as percentage of the maximum response to 50 μM DHPG-stimulated InsP formation in wild type slices. The * indicates statistically significant (P < 0.05) differences in InsP formation following treatment of tissue with either 5 or 50 μm DHPG concentrations between cortical prisms derived from wt and APPswe mice and cortical prisms derived from wt and APPswe/PS1ΔE9 mice that do not express mGluR5. ^#^ Indicates statistically significant (P < 0.05) differences in InsP formation following treatment of tissue with either 5 and 50 μm DHPG concentrations between cortical prisms derived from wt and APPswe that do not express mGluR5.

Aβ oligomers were previously reported to activate mGluR5 and promote mGluR5 clustering at synapses [18]. Therefore, we examined whether the cell surface expression of mGluR5 was altered in APPswe/PS1ΔE9 mice at 12 months of age as compared to wild-type controls. Using coronal brain slices from 12 month old wild type and APPswe/PS1∆E9 mice, we determined total mGluR5 expression, as well as the plasma membrane expression for mGluR5 by performing a cell surface biotinylation assay [32]. We found that the cell surface expression of mGluR5 was increased by 4.4 ± 0.7 fold in APPswe/PS1∆E9 mice when compared with wild-type mice, without a change in total cellular expression of mGluR5 (Figure 4A and B). No mGluR5 expression was observed in mGluR5-/- mouse lines. The observed increase in mGluR5 cell surface expression suggested the possibility that mGluR5-dependent cell signaling in APPswe/PS1∆E9 was increased *in vivo* in response to either glutamate or soluble Aβ oligomers. Therefore, we tested whether InsP formation in cross chopped (prisms) cortical brain slices derived from 12 month old wild-type and APPswe/PS1∆E9 mice, that either express or lack mGluR5 expression was altered following treatment with the Group I mGluR agonist DHPG (5 or 50 μM). We found that despite increased mGluR5 cell surface expression in APPswe/PS1∆E9 mice at 12 months, agonist-stimulated (DHPG) InsP formation was not significantly different between cortical prisms derived from either 12 month old wild-type or APPswe/PS1∆E9 mice (Figure 4C). However, an increase in DHPG-stimulated InsP formation was observed in APPswe/PS1∆E9 mice lacking mGluR5, when compared with wild-type mGluR5 knockout tissue, suggesting that mGluR1 may compensate to some extent for the loss of mGluR5 expression in the APPswe/PS1∆E9 mice (Figure 3C). This might be the consequence of compensatory mGluR1 expression in the APPswe/PS1∆E9 mice [33].

### FMRP expression is increased in APPswe/PS1ΔE9 mice

**Figure 5:**
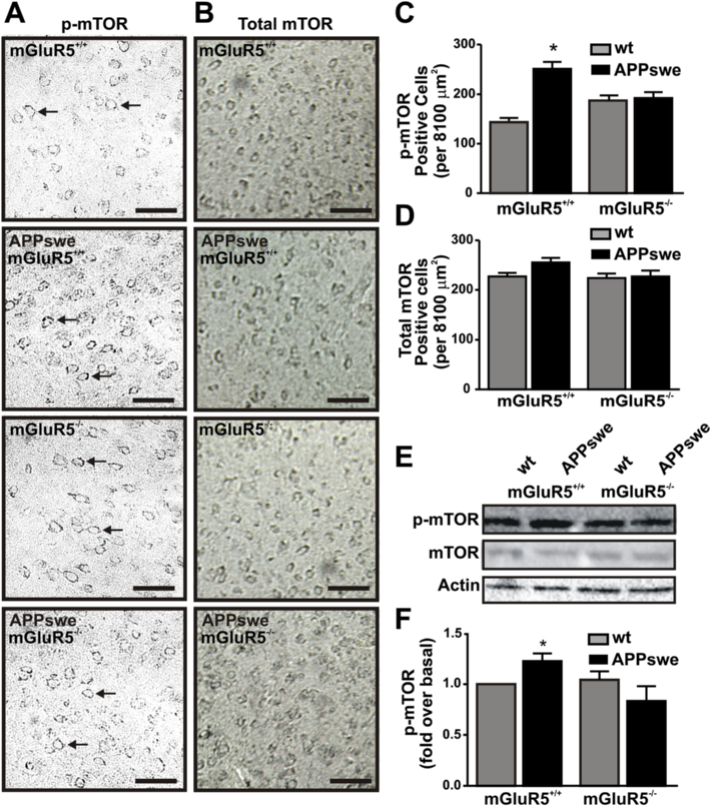
**p-mTOR activation in APPswe/PS1ΔE9 mice.** Shown is immunohistochemical staining **(A)** for p-mTOR (pSer2481) staining in representative fields of coronal tissue from wild type (wt), APPswe/PS1ΔE9 (APPswe), mGluR5-/- and APPswe/mGluR5-/- mice at 12 months of age **(B)** and mTOR (pSer2481) staining in representative fields of coronal tissue from wt, APPswe, mGluR5^-/-^, and APPswe/mGluR5-/- mice at 12 months of age. Scale bar = 100 μm. **(C)** The graph shows the number of p-mTOR positive cells counted 8100 μm^2^ regions of interests in the cortex of 12 month old from wt, APPswe, mGluR5^-/-^, and APPswe/mGluR5-/- mice. Data represents the mean ± SEM of four independent experiments. The * indicates statistically significant (p < 0.05) differences between wt and APPswe mice. **(D)** The graph shows the number of total mTOR positive cells counted 8100 μm^2^ regions of interests in the cortex of 12 month old wt, APPswe, mGluR5-/-, and APPswe/mGluR5-/-. Data represents the mean ± SEM of four independent experiments. Shown in **(E)** is a representative immunoblot for p-mTOR (pSer2481), total mTOR and actin in lysates derived from cortical brain section from wt and APPswe mice that either express mGluR5 (+/+) or do not express mGluR5 (-/-). **(F)** Shown in the graph is the analysis of the mean ± SD for four independent experiments for mTOR phosphorylation in wt and APPswe mouse cellular lysates that either express mGluR5 or do not express mGluR5. The * indicates statistically significant (P < 0.05) differences between mTOR phosphorylations in APPswe as compared to wt mice.

**Figure 6:**
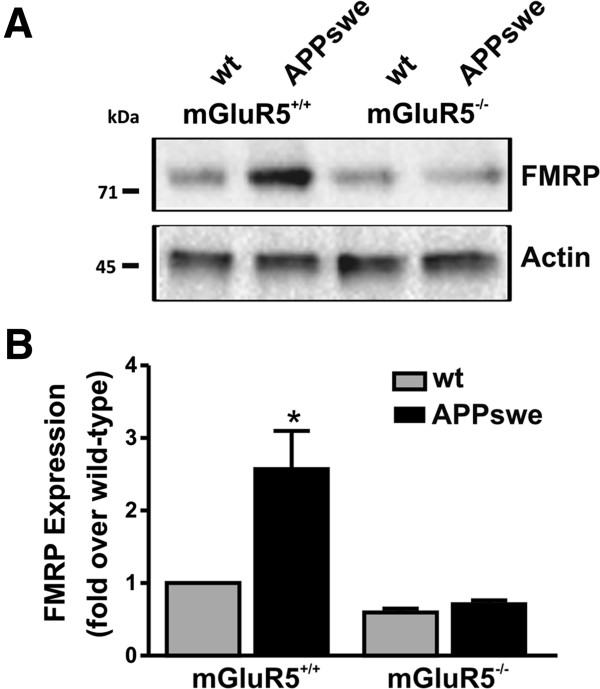
**Increased FMRP protein expression in APPswe/PS1ΔE9 mice*. *(A)** Shown is a representative immunoblot for FMRP expression in wild-type (wt) and APPswe/PS1ΔE9 (APPswe) mice on either mGluR5+/+or mGluR5-/- genetic backgrounds. The data shown in **(B)** represent the mean ± SD of four independent experiments demonstrating increase FMRP protein expression in APPswe mice that is lost following the genetic deletion of mGluR5. The * indicates a statistically (P 0.05) significant differences in FMRP expression between APPswe/PS1∆E9 mice and wild-type mice.

The activity and expression of FMRP, a RNA binding protein involved in the regulation of APP translation and processing, is modulated by mGluR5 [23,24]. Thus, it is possible that mGluR5-activated cell signaling pathways, acting independent of agonist-mediated G protein signaling pathways, might bias mGluR5 signaling, as a consequence of increased cell surface mGluR5 expression in APPswe/PS1∆E9 mice, towards alternative signal transduction pathways. Specifically, mGluR5 activation not only modulates FMRP expression, but regulates FMRP activity via the activation of mTOR [34]. Therefore, we examined whether p-mTOR (p-2481) phosphorylation was increased in cortical brain tissue derived from wild-type and APPswe/PS1∆E9 mice that either express or lack mGluR5 expression at 12 months of age. We found an increase in p-mTOR staining in cortical sections derived from APPswe/PS1∆E9 mice that express mGluR5 when compared to all other genotypes (Figure 5A and C) with no significant alterations in total mTOR staining observed between phenotypes (Figure 5B and D). This corresponded to an 82 ± 17% increase in *in situ* mTOR phosphorylation in cortical tissue slices from APPswe/PS1∆E9 mice at 12 months of age, as compared with wild-type mice. In addition, we examined whether mTOR (p-2481) phosphorylation was increased in brain lysates derived from APPswe/PS1∆E9 mice at 12 months of age and found that mTOR phosphorylation was significantly increased by 22 ± 0.8%, as compared with wild-type mice (Figure 5E and F).Because both mGluR5 and mTOR activation influence FMRP expression and activity, we tested whether FMRP expression was altered in APPswe/PS1∆E9 mice as compared to wild-type mice. We found that FMRP expression was increased by 2.6 ± 0.6 fold in APPswe/PS1∆E9 mice at 12 months of age, as compared to wild-type mice (Figure 6A and B). Genetic deletion of mGluR5 significantly reduced FMRP expression APPswe/PS1∆E9 mice (Figure 6A and B). These observations suggest that mGluR5 activation by Aβ oligomers promotes a positive feedback loop promoting APP cleavage of AD toward the amyloidogenic pathway in APPswe/PS1∆E9 mice.

## Discussion

The neurodegeneration characteristic of AD is associated with Aβ oligomers, which bind to neuronal synapses to both alter synaptic signaling and promote synaptic loss [35-37]_._ We show here, that mGluR5 signaling in double transgenic APPswe/PS1∆E9 mice not only contributes to impaired spatial learning, but also increases the formation of Aβ oligomers and β-amyloid plaques which are the pathophysiological hallmarks of AD. In addition, spatial learning deficits observed in double transgenic APPswe/PS1∆E9 mice are ameliorated by the genetic deletion of mGluR5, which is consistent with the recent report that pharmacological blockade of mGluR5 with the mGluR5-specific antagonist MTEP improved behavioural deficits in APPswe/PS1∆E9 mice [17]. However, we unexpectedly found that mGluR5 signaling may also contribute to the etiology of AD pathology by promoting the formation of Aβ oligomers in APPswe/PS1∆E9 mice, which are considered to be important mediators of neurotoxic signaling at glutamatergic synapses. It is also now recognized that mGluR5 functions as a receptor for both Aβ oligomers [17-19], and PrP^c^ to mediate Aβ oligomer-dependent release of Ca^2+^ from intracellular stores, an event which may be neurotoxic [17]. Aβ oligomers also mediate clustering of mGluR5 at synapses, the consequence of which may contribute to the functional impairment of synaptic activity [18]. Our data suggest that Aβ oligomer interactions with mGluR5 may also function to accelerate Aβ production via the mGluR5- and FMRP-dependent activation of the amyloidogenic pathway. Thus, it appears that in the double transgenic APPswe/PS1∆E9 mouse model of AD, Aβ oligomers initiate a neurodegenerative positive feedback loop that increases mGluR5 activity leading to an increase in Aβ oligomer formation that culminates in AD-like pathology and impaired spatial learning. The genetic deletion of mGluR5 in APPswe/PS1∆E9 mice effectively severs this feedback loop, reducing Aβ oligomer and plaque formation resulting in improved cognitive performance in APPswe/PS1∆E9 mice.

We found that there was no change in swim speed the mouse groups tested suggesting that altered locomotion or anxiety did not account for changes in MWM performance, in the data presented to illustrate learning we showed both path length and escape latency. While an expedient escape latency could be attributed to hyperactivity, the fact that there was no difference in swim speed, and no significant difference in path length, this indicates that the mGluR5 knockout mice learn the task, as well as the wild type animals. We also found no changes in anxiety behaviour in the elevated plus maze. Our results were completely consistent with recent data showing mGluR5 antagonism improved APPswe/PS1∆E9 mouse behaviour in MWM [17]. The previously mGluR5 knockout deficit in the MWM reported by Lu et al. [38] was in mice of undocumented age and did not involve the comparison of aged mice.

The activation of mGluR5 triggers the mRNA binding protein FMRP to release APP repression, promoting its translation [20,23,24]. In fragile X syndrome, this interaction leads to increased cleavage of APP [19,22], suggesting that similar processes may be occurring in AD brain. APP cleavage occurs via a non-amyloidogenic pathway and an amyloidogenic pathway, which is considered to function as an “overflow” pathway activated as a consequence of excessive APP production resulting in increased APP cleavage and the formation of toxic Aβ oligomers [3,39]. Thus, Aβ mediated activation of mGluR5 may result in both increased FMRP expression and APP processing. We propose that the observed increase in FMRP protein expression in APPswe/PS1∆E9 mice represents a compensatory mechanism that is engaged to potentially reduce ectopic/transgenic translational control of APP translation. However, since mTOR activity is also increased in APPswe/PS1∆E9 mice increased FMRP expression levels may be ineffective in reducing APP translation. There is evidence that G protein-coupled receptors regulate γ-secretase activity in a β-arrestin-dependent manner to alter Aβ production [40]. However, the link between mGluR5 signaling and β-arrestin activation is controversial [41]. As a consequence, Aβ oligomer-stimulated mGluR5 activity may function to promote the cleavage of APP via the amyloidogenic pathway.

The degeneration of glutamatergic synapses is associated with disrupted neuronal signaling and synaptic loss has traditionally been associated with alterations in ionotropic NMDA and AMPA receptor signaling [3,42-44]. However, the pharmacological blockade of the NMDAR has not met with success for the treatment of neurodegenerative disease [38]. The recent identification of mGluR5 as a potential disease modifier in AD provides a potential for novel mGluR5 antagonist-based pharmacological interventions for the treatment of AD. mGluR5 blockade was effective in improving spatial learning in APPswe/PS1∆E9 mice and we reported that mutant huntingtin protein aggregates are significantly reduced in a mutant Huntingtin knock-in model that lacks mGluR5 expression [12]. Taken together these observations suggest a generalized potential for mGluR5 antagonists in the treatment of neurodegenerative disease.

In summary, we show that mGluR5, which functions as a membrane bound receptor scaffolds for both PrPc and Aβ oligomers [17-19], functions to transduce Aβ oligomer-mediated signals as alterations in intracellular Ca^2+^ signaling, as well as FMRP/Aβ mediated pathological signaling in the APPswe/PS1∆E9 mouse model of AD. Although these studies provide additional evidence establishing mGluR5 as a potentially effective drug target to antagonize AD progression, additional pharmacological studies will be required in non-human primates to validate the utility of mGluR5 antagonists for the treatment of AD patients.

## Materials and methods

### Materials

Sulfo-NHS-SS-biotin and Neutravidin were purchased from Thermo Scientific (Waltham, MA, USA. Mouse anti-FMRP antibody was purchased from Abcam (Cambridge, UK). DHPG was purchased from Tocris Bioscience (Bristol, UK). Myo-[^3^H]-inositol was purchased from Perkin Elmer (Waltham, MA, USA). Dowex I-X8 resin, rabbit anti APP antibody, rabbit anti-phospho mTOR (pSer2481) antibody and rabbit anti-mTOR antibody were purchased from Sigma-Aldrich (St. Louis, MO, USA).Goat anti-rabbit secondary antibody was purchased from BioRad (Hercules, CA, USA). Aggregated Aβ human ELISA kit and rabbit Aβ peptide antibody were purchased from Invitrogen (Carlsbad, CA, USA). Rabbit anti-mGluR5 was purchased from Millipore (Billerica, MA, USA). Finally Vector Elite ABC kit rabbit and mouse, and Vector SG substrate was purchased from Vector laboratories. All other biochemical reagents were purchased from Sigma-Aldrich (St. Louis, MO, USA).

### Mouse model

STOCK B6C3-Tg (APPswe/PSEN1∆E9)85Dbo/J mice that carry the human APP with Swedish mutation and the DeltaE9 mutation of the human presenilin 1 gene and mGluR5 knockout mice B6;129-Grm5^tm1Rod^/J (*mGluR5*^*-/-*^) were purchased from Jackson Laboratory (Bar Harbor, ME) [38,45]. APPswe/PS1∆E9/mGluR5^-/-^ mice were generated by crossing APPswe/PS1∆E9 mice with a C57/Bl6 background, with mGluR5-/- C57/Bl6 females. Offspring were tail snipped and genotyped using PCR with primers specific for the APP sequence and primers specific for mGluR5. The following genotypes were generated and used for experiments APPswe/PS1ΔE9/mGluR5^-/-^, APPswe/PS1ΔE9/mGluR5^+/+^, APPswe/PS1ΔE9^-/-^/mGluR5^-/-^ and wild type littermate controls. Animals were housed in an animal care facility in cages of 2 or more animals, and were maintained on a 12 hour light/12 hour dark cycle at 24°C. Mice received food and water *ad libitum*. Mice were aged to 9 and 12 months before use in a battery of behavioural and biochemical experiments. All animal experiments were conducted in accordance with the University of Western Ontario animal care committee.

### Morris water maze

Any Maze software connected to a video camera was used to track the activity of animals within the maze. The maze was a white opaque plastic pool (120 cm in diameter), filled with water and maintained at 25°C to prevent hypothermia. A clear escape platform (10 cm diameter) was placed 25 cm from the perimeter, hidden 1 cm beneath the surface of the water. Visual cues were placed on the walls in the room with the maze. *Acquisition phase (day 1–4)*: Mice were trained over 4 consecutive days, with 4 trials per day with 15 min intervals between trials. Mice were randomly started from 4 equally spaced points around the pool, across each of the 4 daily trials. Animals were given 90 s to find the escape platform, if they failed to do so they were guided to the platform where they remained for 30 s, before being returned to their home cage. Swim speed and escape latency were recorded and statistical analysis performed using Graph Pad Prism. *Probe trial (day 5)*: the probe trial is performed to assess spatial memory. This is a singular 60 s trial, in which the platform is removed and mice are allowed to swim freely in the pool. Time spent in the target quadrant was recorded and statistical analysis performed using Graph Pad Prism software.

### Open field

VersaMax animal activity monitors (AccuScan Instruments Inc, Columbus, OH, USA) were used to measure locomotor activity. Experiments were performed during the light cycle of the mice. Mice were allowed to explore open field boxes (20 × 20 cm Plexi-glass boxes) for 120 min during which time movement was measured at 5 min intervals using beam breaks converted to cm. Measurements of total distance travelled, center time and number of rearings were calculated and statistical analysis performed using Graph Pad Prism software.

### Elevated plus maze

Mice were individually placed in one of the enclosed arms of a plus-maze and the behavior of the animals was recorded over a 5 min period by a ceiling-mounted video camera. The amount of time spent in each of the arms, the number of arm entries (an arm entry was defined as all four of the paws being placed in an arm of the plus-maze). The elevated plus-maze had two arms enclosed by 21 cm high walls; whereas the remaining two arms were open (arms were 24.8 cm long × 7.7 cm wide). The maze was situated in a dimly lit room, such that the closed arms were darkened, whereas open arms were somewhat illuminated. All behavioural experiments were blinded.

### Determination of β amyloid oligomer concentration by sandwich ELISA

Brains were dissected into right and left hemisphere, with one hemisphere used to analyse oligomeric Aβ. Quantification of Aβ oligomers, in 12 month old fresh mouse brains, was performed using a sandwich ELISA kit (KHB3491, Invitrogen) according to manufacturer’s instructions. Briefly, wild-type, APPswe/PS1ΔE9/mGluR5^-/-^, APPswe/PS1ΔE9/mGluR5^+/+^, APPswe/PS1ΔE9^-/-^/mGluR5^-/-^ mouse brains were homogenized in Tris Buffered Saline (25 mM Tris–HCl, pH 7.4; 150 mM NaCl) supplemented with protease inhibitor cocktail (1 mM AEBSF, and10 μg/ml aprotinin of both leupeptin). Brain homogenates were centrifuged at 100,000 × *g at* 4°C for 1 hour. The supernatant was then diluted 1:10 before carrying out the ELISA, which was performed in triplicate, measuring only Aβ oligomers, as detailed in the manufacturer's protocol. Protein was quantified using the Bradford protein assay (BioRad). The final Aβ values were determined following normalization to total protein levels.

### β amyloid immunohistochemistry

Brains were dissected into left and right hemisphere, with one hemisphere used for histology. Brains were coronally sectioned to include both the cortex and hippocampus. Immunohistochemistry was performed on 40 μm free floating sections using a peroxidase based immunostaining protocol. In brief, endogenous peroxidase activity was quenched using 0.1% hydrogen peroxide, after which the membranes were permeabilized using 1% triton X100 in TBS. Non-specific binding was blocked using 1.5% normal goat serum, followed by incubation in primary antibody for Aβ (1:200, rabbit polyclonal, Invitrogen) overnight at 4°C. Sections were washed in 1xTBS, and then incubated in secondary antibody (biotinylated goat anti-rabbit, 1:400, Vector Elite ABC kit rabbit, Vector Laboratories) for 90 min at 4°C. Finally sections were incubated in an avidin biotin enzyme reagent (Vector Elite ABC kit rabbit, Vector Laboratories). Immunostaining was visualised using a chromogen (Vector SG substrate, Vector Laboratories). Sections were mounted on slides and visualized using a Zeiss LSM-510 META multiphoton laser scanning microscope with a Zeiss 10× lens, representative 900 μm × 900 μm areas of cortex (4 regions of interest) and hippocampus (2 regions of interest) were imaged for analysis. The number of Aβ positive puncta per image was counted using the cell counter tool in Image J (NIH, USA).

### Cell surface biotinylation

Cell surface biotinylation was performed as previously described^11^. 350 μm coronal brain slices from wild-type, APPswe/PS1ΔE9/mGluR5^-/-^, APPswe/PS1ΔE9/mGluR5^+/+^, APPswe/PS1ΔE9^-/-^/mGluR5^-/-^mice were prepared using a vibratome system (Leica). Slices were recovered in KREBS buffer (127 mM NaCl, 2 mM KCl, 10 mM glucose, 1.2 mM KH_2_PO_4_, 26 mM NaH_2_CO_3_, 1 mM MgSO_4_, 1 mM CaCl_2_, pH 7.4) continuously gassed with 95%O_2_/5%CO_2_ for 30 min at 37°C. Slices were transferred to tubes and biotinylated for 1 hr in 1.5 mg/ml sulfo-NHS-SS-biotin (Thermo Scientific) for 1 h on ice. Slices were washed and biotinylation quenched with 100 μM glycine in HBSS for 30 min on ice. Following washes in HBSS, tissue was lysed in RIPA buffer (0.15 M NaCl, 0.05 M Tris–HCl, pH 7.2, 0.05 M EDTA, 1% Nonidet P40, 1% Triton X-100, 0.5% sodium deoxycholate, 0.1% SDS) containing protease inhibitors (1 mM AEBSF and 10 μg/ml of both leupeptin and aprotinin), lysates were then polytroned until homogenous. Biotinylated proteins were then precipitated on NeutrAvidin beads using equivalent amounts of cellular protein for each sample. Biotinylated proteins were subjected to SDS-PAGE and immunoblotted with Rabbit polyclonal mGluR5 antibody (1:1000, dilution), as described below.

### Immunoblotting

Acute brain slices prepared, as described above, and recovered for 90 min at 37°C in KREBS continuously gassed with 95%O_2_/5%CO_2_. Slices were then transferred to tubes, gassed with 95%O_2_/5%CO_2_ before being capped tightly and left to further recover at 37°C for 30 min. Slices were flash frozen with 95% ethanol in a bath of dry ice and lysed in RIPA buffer. 100 μg of total cellular protein for each sample were subjected to SDS-PAGE, followed by electroblotting onto nitrocellulose membranes. Membranes were blocked with 10% milk in wash buffer (150 mM NaCl, 10 mM Tris–HCl, pH 7.0, and 0.05% Tween 20) for 1 h and then incubated with rabbit anti-APP (1:4000), rabbit anti-p-mTOR (pSer2481, 1:1000), rabbit anti-mTOR (1:1000), mouse anti-FMRP (1:1000) or rabbit anti-actin (1:10000) antibodies in wash buffer containing 3% milk overnight. Membranes were rinsed three times with wash buffer and then incubated with secondary horseradish peroxidase-conjugated goat anti-rabbit or anti-mouse IgG diluted 1:10000 in wash buffer containing 3% skim milk for 1 h.

### Inositol phosphate formation assay

[^3^H]Inositol phosphate formation in cortical slices was performed, as previously described^11^. Dissected cortices from age-matched wild-type, APPswe/PS1ΔE9/mGluR5^-/-^, APPswe/PS1ΔE9/mGluR5^+/+^, APPswe/PS1ΔE9^-/-^/mGluR5^-/-^ mouse brains were cross-chopped using a McIlwain tissue chopper. The cortical prisms were recovered by incubation in KREBS buffer after being gassed with 95%O_2_/5%CO_2_ in a shaking water bath at 37°C for 30 min. 40 μl of gravity-packed prisms were transferred in duplicate to tubes, gassed, and incubated with 1 μCi/ml *myo*-[^3^H]inositol in a shaking bath at 37°C for 90 min. The prisms were gassed again and incubated in a shaking water bath at 37°C for 15 min in 10 mM LiCl followed by stimulation with 5 μM or 50 μM DHPG for 20 min. The reaction was stopped by adding 900 μl of a 2:1 mix of chloroform:methanol incubated for 15 min at room temperature, followed by adding 300 μl of chloroform. The total [^3^H]inositol incorporated into slices was determined by counting the radioactivity present in the hydrophobic layer. 700 μl of aqueous layer was added to Dowex 1-X8 (formate form) 200–400 mesh anion exchange resin in columns. Columns were washed three times with water followed by 2 washes with 60 mM ammonium formate. Samples were eluted with 200 mM ammonium formate and 0.1 M formic acid into scintillation vials containing scintillation fluid. [^3^H]Inositol phosphate formation was determined by liquid scintillation using a Beckman LS 6500 scintillation system.

### p-TOR and mTOR immunohistochemistry

Brains were coronally sectioned to include both the cortex and hippocampus. Immunohistochemistry was performed on 40 μm free floating sections using a peroxidase based immunostaining protocol. In brief; endogenous peroxidase activity was quenched using 0.1% hydrogen peroxide, after which the membranes were permeabilized using 1% triton X100 in TBS. Non-specific binding was blocked using 1.5% normal goat serum, followed by incubation in primary antibody for p-mTOR (1:200 diltion, rabbit polyclonal pSer2481 antibody) and mTOR (1:200, rabbit polyclonal antibody) overnight at 4°C. Sections were washed in 1xTBS, and then incubated in secondary antibody (biotinylated goat anti-rabbit, 1:400, Vector Elite ABC kit rabbit, Vector Laboratories) for 90 min at 4°C. Finally sections were incubated in an avidin biotin enzyme reagent (Vector Elite ABC kit rabbit, Vector Laboratories). Immunostaining was visualised using a chromogen (Vector SG substrate, Vector Laboratories). Sections were mounted on slides and visualized using a Zeiss LSM-510 META multiphoton laser scanning microscope with a Zeiss 10× lens, representative 900 μm × 900 μm areas of cortex (4 regions of interest) and hippocampus (2 regions of interest) were imaged for analysis. The number of p-mTOR and mTOR positive cells per image was counted using the cell counter tool in Image J (NIH, USA).

### Data analysis

Means ± SEM are shown for the number of independent experiments indicated in *Figure Legends*. GraphPad Prism software was used to analyze data for statistical significance and for curve fitting. Statistical significance was determined by analysis of variance (ANOVA) followed by post-hoc testing.

## Abbreviations

AD: Alzheimer’s disease; APP: Amyloid precursor protein; Aβ: β-amyloid; PrP^c^: Cellular prion protein; FMRP: Fragile X mental retardation protein; InsP: Inositol phosphate; mGluR5: Metabotropic glutamate receptor 5.

## Competing interests

The authors declare that they have no competing interests.

## Authors’ contribution

AH: performed the behavioural experiments, beta amyloid analysis, immunohistochemistry, and biochemistry, data analysis and writing of the manuscript. JLE: performed cell surface biotinylation and IP experiments. RAD: assisted with a number of the behaviour experiments. SPC: contributed to the writing of the manuscript. SSGF: Principle investigator, contributed to the experimental design, data analysis and writing of the manuscript. All authors read and approved the final manuscript.

## Supplementary Material

Additional file 1: Figure S1 Time spent in each of the quadrants of the Morris Water Maze for each of the mouse genotypes tested and 12 months of age. Click here for file
