# Supplementary figures and images for: Metabotropic glutamate receptor 5 knockout reduces cognitive impairment and pathogenesis in a mouse model of Alzheimer's disease
Source: Mol Brain. 2014 May 29;7:40. doi: 10.1186/1756-6606-7-40 (PMC4050478; doi:10.1186/1756-6606-7-40)

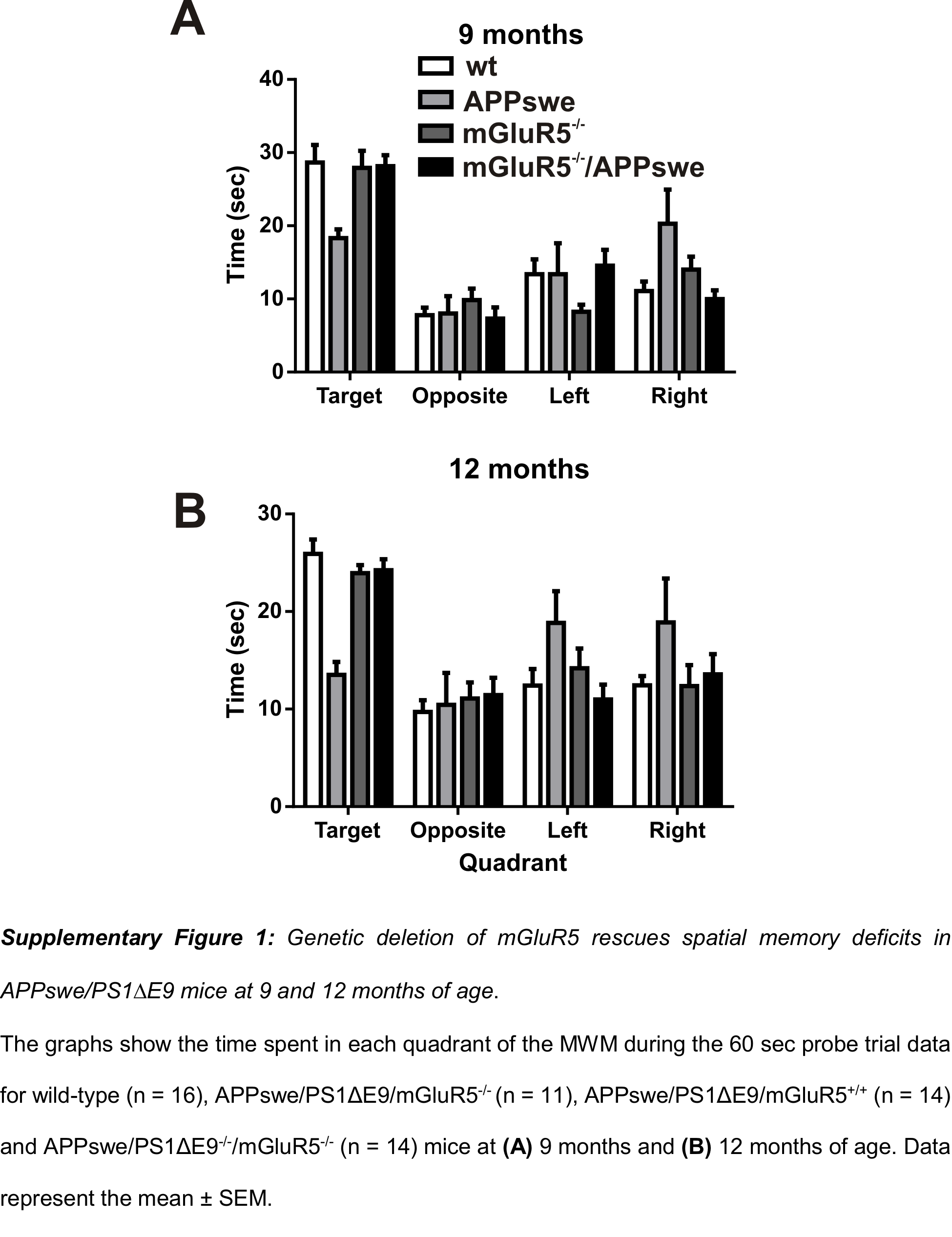

Supplement: Additional file 1: Figure S1 — Time spent in each of the quadrants of the Morris Water Maze for each of the mouse genotypes tested and 12 months of age. [file 1756-6606-7-40-S1.tiff]
